# AI in Hepatology: Revolutionizing the Diagnosis and Management of Liver Disease

**DOI:** 10.3390/jcm13247833

**Published:** 2024-12-22

**Authors:** Sheza Malik, Rishi Das, Thanita Thongtan, Kathryn Thompson, Nader Dbouk

**Affiliations:** 1 Department of Internal Medicine, Rochester General Hospital, Rochester, NY 14621, USA; sheza.malik@rochesterregional.org; 2 Division of Digestive Diseases, Emory University School of Medicine, Atlanta, GA 30322, USA; rishi.das@emory.edu (R.D.); thanita.thongtan@emory.edu (T.T.); 3 Department of Medicine, Emory University School of Medicine, Atlanta, GA 30322, USA; kathryn.monti.thompson@emory.edu; 4 Emory Transplant Center, Emory University School of Medicine, Atlanta, GA 30322, USA

**Keywords:** artificial intelligence, machine learning, deep learning, cirrhosis, hepatocellular carcinoma

## Abstract

The integration of artificial intelligence (AI) into hepatology is revolutionizing the diagnosis and management of liver diseases amidst a rising global burden of conditions like metabolic-associated steatotic liver disease (MASLD). AI harnesses vast datasets and complex algorithms to enhance clinical decision making and patient outcomes. AI’s applications in hepatology span a variety of conditions, including autoimmune hepatitis, primary biliary cholangitis, primary sclerosing cholangitis, MASLD, hepatitis B, and hepatocellular carcinoma. It enables early detection, predicts disease progression, and supports more precise treatment strategies. Despite its transformative potential, challenges remain, including data integration, algorithm transparency, and computational demands. This review examines the current state of AI in hepatology, exploring its applications, limitations, and the opportunities it presents to enhance liver health and care delivery.

## 1. Introduction

**Figure 1:**
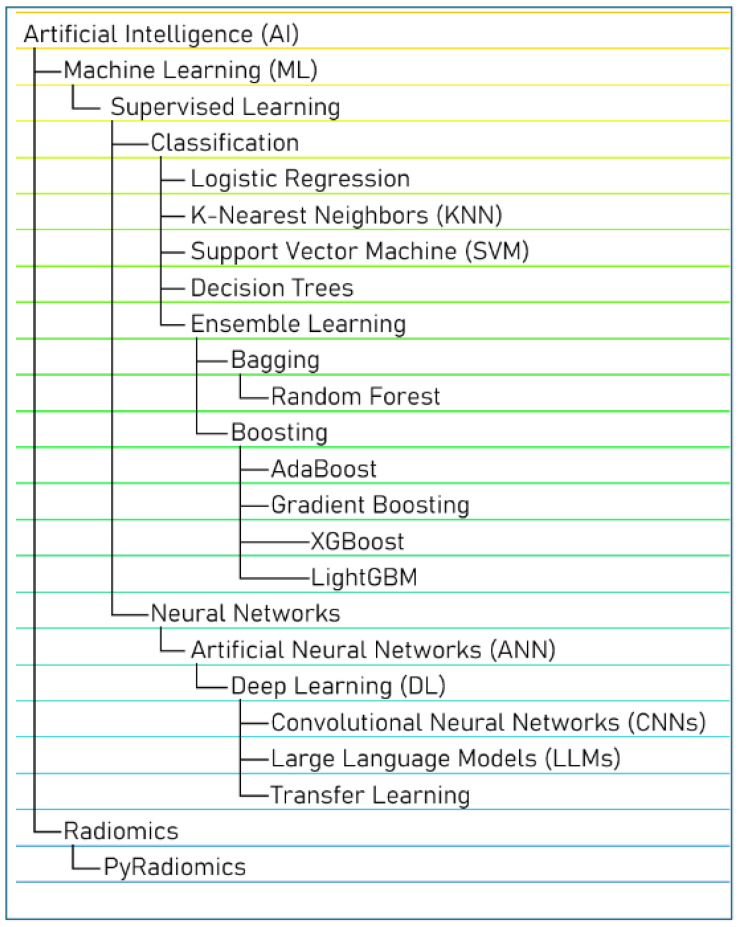
AI hierarchy.

As artificial intelligence (AI) has continued to evolve, its integration into various medical subspecialties has grown significantly, offering innovative approaches to diagnosis, treatment, and disease management [1,2,3,4]. Hepatology is no exception. With the rising global burden of liver conditions such as metabolic dysfunction-associated steatotic liver disease (MASLD) [5,6], the need for efficient and accurate tools for early detection and management has never been more critical. Hepatocellular carcinoma (HCC), a leading cause of cancer-related deaths worldwide, further underscores the urgent need for innovative tools to aid in early detection and personalized management [7,8]. AI technologies have emerged as powerful tools, leveraging vast datasets to improve clinical decision making and patient outcomes. Machine learning (ML), a subfield of AI, leverages diverse algorithms to enhance predictive capabilities and decision making [9]. ML techniques share the following common goal: systematically organizing inputs into features, labels, clusters, or neural networks to improve prediction accuracy and refine outcomes across successive iterations. ML models span a spectrum of human involvement, from supervised learning, where labeled data guide training, to unsupervised learning, which identifies patterns in unlabeled data. Reinforcement learning, another subset, involves algorithms learning optimal actions through trial and error. Natural Language Processing (NLP) enables the automated extraction of relevant clinical information from text, which can be utilized for tasks like clinical surveillance or as input features for other predictive modeling algorithms. Deep learning (DL), a specialized branch of ML, mimics human brain processing by employing multi-layered neural networks with minimal human supervision, but requires significant computational power [10]. These approaches collectively drive advancements in AI applications, transforming complex data into actionable insights in medicine and beyond. Figure 1 depicts the hierarchy of AI models, while Table 1 provides an overview of various commonly employed models.

A review of the current literature highlights AI’s applications in diagnosing and managing a wide range of liver diseases, including autoimmune hepatitis (AIH), primary biliary cholangitis (PBC), primary sclerosing cholangitis (PSC) [11], MASLD [12,13,14,15], and viral hepatitis [16,17,18]. Beyond diagnosis, AI shows promise in predicting disease progression, such as advanced fibrosis and cirrhosis, and plays a growing role in hepatocellular carcinoma (HCC) diagnosis and management [19,20,21].

Despite these promising developments, challenges persist. Data heterogeneity, ethical considerations, and the complexity of integrating AI into clinical workflows are significant obstacles. Model interpretability remains a key issue, as clinicians must trust and understand AI-driven recommendations. Addressing these limitations will be essential for fully realizing AI’s potential in hepatology.

The aim of this review article is to explore the transformative applications of artificial intelligence (AI) in hepatology, focusing on its potential to enhance diagnosis, treatment, and disease management in liver-related conditions. By examining recent advancements in AI-driven imaging, predictive modeling, and personalized medicine, this article seeks to provide a comprehensive understanding of how these technologies can be integrated into clinical practice. Additionally, it will highlight the challenges, ethical considerations, and future directions for AI in hepatology, offering valuable insights for researchers, clinicians, and policymakers aiming to leverage AI for improved patient outcomes.

## 2. Applications of AI in the Diagnosis and Management of Cirrhosis and Its Complications

AI has shown promise in improving the accuracy of diagnosing and managing cirrhosis and its complications through advanced imaging techniques, ML algorithms, and biomarker analysis. While these developments suggest potential benefits, critical evaluation reveals limitations in generalizability, reliance on specific datasets, and a lack of external validation. This section examines the current applications of AI in this area, emphasizing both advancements and areas needing further refinement. Table 2 summarizes the studies referenced in this section.

### 2.1. Detecting Advanced Fibrosis and Cirrhosis

Chang et al. assessed multiple ML models, including logistic regression, random forest (RF), and artificial neural networks (ANNs) for predicting the fibrosis stages in 1370 MASLD patients, showing a superior predictive accuracy compared to conventional tools such as Fibroscan and FIB-4 [22]. While promising, the study relied heavily on specific demographic and clinical variables, raising concerns about its applicability to diverse populations. Similarly, Fan et al. demonstrated a high diagnostic accuracy with ML algorithms that integrated liver stiffness measurements and the aMAP score—a composite index including age, gender, albumin, bilirubin, and platelet count—for diagnosing fibrosis and cirrhosis in 946 MASLD patients from China and the United States. However, the applicability of these findings is constrained by their lack of external validation across more diverse populations [23]. In another study, Anushiravani et al. used RF algorithms to develop the FIB-6 index, which demonstrated a high sensitivity and specificity in ruling out cirrhosis in HCV patients. The FIB-6 index was then validated in a cohort of 2472 biopsy-proven MASLD patients from Egypt, Iran, Saudi Arabia, Greece, Turkey, and Oman and outperformed other non-invasive indices [24]. Despite the FIB-6 index’s strong performance, its reliance on biopsy-proven data from specific cohorts limits its generalizability to other etiologies and regions.

Several studies highlight the advantages of the Light Gradient Boosting Machine algorithm (LightGBM) in identifying advanced fibrosis and cirrhosis. Light GBM’s memory-efficient structure and rapid processing capabilities make it particularly well-suited for analyzing large-scale datasets, often encountered in clinical and population-level studies. For example, Wang et al. constructed a model using Light GBM to predict cirrhosis in patients with chronic HBV, leveraging platelet count and bile acid levels. This model demonstrated a superior predictive accuracy compared to conventional non-invasive measures [25]. Orhanbulucu et al. employed ensemble learning techniques, including LightGBM, to predict chronic liver disease (CLD) [26]. In this study, LightGBM achieved the highest accuracy (98.8%) among various boosting algorithms, including RF and AdaBoost. Similarly, Kuzhippallil et al., used LightGBM to predict CLD alongside other algorithms like XGBoost and stacking. After feature selection and outlier elimination, LightGBM demonstrated a competitive accuracy, reinforcing its utility as a disease prediction model [27]. LightGBM’s strong performance in predicting cirrhosis and CLD highlights its potential for integration into clinical workflows, potentially replacing or complementing traditional non-invasive methods. Further studies should focus on external validation in diverse populations, enhancing model interpretability, and addressing barriers to clinical implementation.

ML models incorporating imaging have also shown significant potential in predicting cirrhosis. Mazumder et al. trained an automated liver segmentation model using a dataset of 1590 CT scans, combining 3D-U-Net and Google’s DeepLabv3+ algorithms. By integrating routine lab values and demographic data, they achieved an AUC of 0.85 in a validation cohort of 352 patients, including 72 liver transplant recipients [28]. Similarly, Nowak et al. applied deep transfer learning with a ResNet50 CNN pre-trained on the ImageNet archive. Their model outperformed a board-certified radiologist in detecting cirrhosis among 713 patients [29].

Deep learning (DL) has also been applied to MRI data to differentiate alcohol-related cirrhosis from non-alcohol-related cases. Luetkens et al. demonstrated that ResNet50 achieved the highest performance in a cohort of 465 patients, with an accuracy of 75% and an AUC of 0.82 [30]. Wei et al. used Pyradiomics software to extract radiomic features from the MRI sequences of 280 patients with CLD, enabling effective fibrosis staging and inflammation grading [31].

ML models derived from other imaging modalities have also shown promise. Destrempes et al. combined ultrasound and shear wave elastography data in an ML model for 82 CLD patients, achieving a better accuracy in identifying steatosis, inflammation, and fibrosis than elastography alone [32]. Similarly, Meng et al. utilized ultrasound and elastography data from 618 patients with diabetes and MASLD. Their approach identified 25 fibrosis-related radiomic features and highlighted 5 of these as the most predictive, demonstrating the utility of ML in fibrosis prediction [33].

These studies highlight the promise of integrating advanced imaging techniques with ML algorithms to improve non-invasive cirrhosis diagnostics. However, their broader applicability in clinical practice is limited by several factors. Many of these studies are retrospective and rely on relatively small sample sizes, with limited validation across diverse populations. Additionally, the complexity of the preprocessing and feature extraction steps in some algorithms poses practical challenges, potentially hindering usability in routine clinical workflows. To address these limitations, larger, multicenter studies are essential to validate these models across varied demographic and clinical settings. Simplifying preprocessing requirements and embedding these models directly into radiological workflows could further enhance their accessibility and adoption in clinical practice.

### 2.2. Detecting Clinically Significant Portal Hypertension (CSPH) and High-Risk Esophageal Varices

The early detection of CSPH is critical, as timely interventions can prevent progression to decompensation. While hepatic venous pressure gradient (HVPG) measurement remains the gold standard for diagnosing portal hypertension, its invasive nature and limited availability outside tertiary care centers underscore the need for reliable non-invasive alternatives.

Several studies have explored the potential of ML to address this gap. Bosch et al. developed an ML model using trichrome-stained liver biopsy samples from patients with cirrhosis due to MASLD. This model identified CSPH with AUCs of 0.85 and 0.76 in the training and test phases, respectively [34]. Similarly, Yu et al. applied an automated CT radiomics HVPG quantitative model (aHVPG), which demonstrated a superior performance compared to other non-invasive measures, including elastography, in assessing portal hypertension severity [35]. Noureddin et al. leveraged ML to create a scoring system based on septa, nodules, and fibrosis (SNOF) derived from liver biopsies in MASLD-related cirrhosis, achieving a high predictive accuracy for CSPH [36], while Reinis et al. employed ML models based on common lab parameters—bilirubin, platelet count, and international normalized ratio (INR)—to predict CSPH in patients with compensated advanced chronic liver disease (cACLD), reporting an AUC of 0.775 [37].

The development of esophageal varices is one of the earliest indicators of CSPH. The early identification of varices, especially those at a high risk of bleeding, enables timely interventions like banding and the initiation of non-selective beta blocker therapy to lower bleeding risk. ML has proven to be beneficial in this context as well. Liu et al. demonstrated the utility of XGBoost in predicting variceal rebleeding. Similarly, Agarwal et al. used XGBoost to predict bleeding risk in cACLD, providing a high-accuracy, non-invasive alternative to endoscopic evaluations [38,39]. Bayani et al. further enhanced non-invasive diagnostic capabilities by integrating elastography with clinical and laboratory data in an ensemble learning model, achieving an AUC of 0.87 and outperforming traditional methods like the platelet-to-spleen ratio [40]. Gao et al. created an imaging-based ML model combining endoscopic images and structured data, surpassing traditional clinical scores in predicting variceal bleeding in cirrhosis patients [41]. Jin et al. introduced an AI-driven tool for measuring the esophageal varix diameter via endoscopy, enabling accurate risk assessment without direct visualization [42]. Yan et al. developed a radiomics model leveraging portal venous-phase enhanced CT images, achieving a superior diagnostic accuracy compared to the Baveno VI criteria [43].

CNNs have also been explored in this domain. Hou et al. created a neural network to predict the 1-year risk of esophagogastric variceal bleeding based on 12 independent risk factors, outperforming conventional indices [44]. Similarly, Chen et al. evaluated ENDOANGEL, a deep convolutional neural network trained on 14,718 endoscopic images, which excelled in diagnosing gastroesophageal varices (EVs and GVs) and predicting rupture risk, outperforming endoscopists in several critical areas [45]

While these studies are promising, they come with notable limitations. For example, models like those developed by Bosch and Noureddin rely on liver biopsy data, an invasive method that hinders broad applicability. Others depend on advanced imaging or computational resources, restricting their use in settings with limited resources. To improve clinical utility, future research should focus on developing models based on easily accessible, non-invasive data and ensure validation across diverse, multicenter populations to enhance robustness and generalizability.

Similarly, while the potential of AI in variceal management is evident, several challenges must be addressed. Many models depend on endoscopic visualization, advanced imaging techniques, or significant computational resources, limiting their feasibility in resource-constrained environments. Additionally, the retrospective design and single-center focus of most studies raise concerns about their generalizability. Validation in larger, multicenter cohorts is crucial to strengthen the reliability of these findings.

### 2.3. Management of Decompensated Liver Disease

Machine learning (ML) models show promise in improving the management of ascites by offering non-invasive and personalized approaches. Wurstle et al. developed a model incorporating 34 commonly available clinical data points to identify patients with infected ascites. This model demonstrated high negative predictive values across a range of pre-test probabilities, suggesting that it could serve as a reliable non-invasive alternative to paracentesis [46]. Similarly, Hatami et al. employed ML models, including k-nearest neighbors (KNNs), support vector machines (SVMs), and neural networks, to predict ascites grades using routine clinical data. Among these, the KNN model achieved the highest accuracy at 94%, highlighting ML’s potential for effectively predicting ascites severity [47].

ML models are also increasingly being utilized to predict outcomes in patients with cirrhosis and hepatic encephalopathy (HE), offering potential advancements in clinical decision making. In a retrospective study using the MIMIC-IV database, Yang et al. developed and validated ML models to predict early mortality in HE patients. Among the models evaluated, the artificial neural network (NNET) demonstrated the highest predictive accuracy, achieving an AUC of 0.837. The NNET model also outperformed traditional clinical scores, such as MELD and MELD-Na. This suggests that NNET could effectively identify HE patients at a high risk for mortality, potentially guiding aggressive treatment strategies and early liver transplant referrals. However, external, prospective validation is necessary to confirm its applicability across diverse patient populations [48].

Similarly, Zhang et al. employed weighted ML algorithms to predict mortality risk in cirrhotic patients with HE. Their weighted random forest model demonstrated a high sensitivity and specificity, with an AUC of 0.82, and effectively differentiated between high-risk and uncomplicated HE cases. These findings further highlight the promise of ML in enhancing outcome prediction and tailoring interventions for patients with complex liver disease [49].

While these studies underscore the potential benefits of ML in managing both ascites and hepatic encephalopathy (HE), important limitations remain. Models like the one proposed by Wurstle et al. heavily depend on the quality and completeness of input data, which can vary across different healthcare settings. Moreover, these models need validation in larger, more diverse patient populations to ensure their generalizability. Another limitation is the computational complexity and resource demands of some models, which may pose challenges in resource-limited or community settings. Future research should focus on developing robust, easily implementable models across various clinical environments. Similarly, for managing HE, the reliance on retrospective data introduces a risk of selection bias, potentially limiting the generalizability of findings to broader patient populations. While the NNET and weighted random forest models showed strong performance metrics, their practical implementation in clinical settings may be hindered by their computational complexity and the need for comprehensive input data. Prospective validation in multicenter, diverse patient populations is crucial to assess the robustness and reproducibility of these models.

## 3. Applications of AI in the Diagnosis and Management of HCC

HCC represents a significant global health challenge due to its high mortality rate and complex management requirements. HCC is the most common form of primary liver cancer and the third leading cause of cancer-related death worldwide [50]. The high mortality rate associated with HCC is largely due to late-stage diagnosis and the limited effectiveness of conventional treatments, with a 5-year survival rate of only 15% for unresectable disease [51]. Traditional predictive and risk stratification models for HCC rely on demographic data, laboratory findings, and elastography to generate risk profiles ranging from low to high using multivariate regression modeling [52,53,54]. The current guidelines for HCC are informed by its natural history, lesion detection sensitivity, and the cost-effectiveness of screening intervals. However, advancements in big data processing and the refinement of modern algorithms offer exciting possibilities for improving risk stratification, diagnostic accuracy, and tailored therapeutic approaches. In the following sections, we will outline efforts to implement ML methods to identify the risks of developing HCC among patients with CLD; diagnose HCC with imaging, pathology, and genomic markers; and predict treatment response. Table 3 summarizes the studies referenced in this section.

### 3.1. HCC Risk Stratification

ML models have demonstrated effectiveness in predicting the risk of HCC in patients with cirrhosis. For instance, Singal et al. developed an ML model using RF and a regression model using a cohort of 442 patients with cirrhosis enrolled in an HCC surveillance program. They found that their ML model outperformed the regression model in identifying patients at a high risk for developing HCC, showing a superior predictive capability [55].

Similarly, Kim et al. used gradient boosting algorithms in a cohort of 6051 Korean patients with HBV to create an ML model. This model was compared with other predictive models across both Korean and Caucasian validation cohorts. The ML model demonstrated superiority in predicting HCC risk in these diverse populations, outperforming various scoring indices [56].

In a large retrospective cohort of 4242 patients with HBV and HCV, Sato et al. evaluated multiple ML methods for HCC prediction. They found that gradient boosting achieved the highest accuracy (87.34%; AUC = 0.940), followed closely by RF (86.08%; AUC = 0.923), outperforming logistic regression (79.74%; AUC = 0.866). This study highlighted the advantage of decision-tree-based models like RF over neural networks for HCC prediction [57]. A similar study from Hong Kong by Wong et al. compared multiple ML models for predicting the HCC risk among 124,006 patients with HBV and HCV. Ridge regression (AUROC = 0.844, sensitivity = 0.90, specificity = 0.90 with dual cutoffs) and RF (AUROC = 0.837, sensitivity = 0.90, specificity = 0.910 with dual cut-offs) produced the highest model accuracy in the validation cohort [58].

More recently, Lin et al. used a sample of 5155 Korean patients with multiple etiologies of CLD to develop an ML-based model named SMART-HCC. This model incorporated elastography measurements and was validated in both Korean and Caucasian cohorts, showing a superior performance compared to multiple other traditional predictive models. SMART-HCC successfully stratified patients into low-, intermediate-, and high-risk groups, with a significantly higher incidence of HCC in the high-risk group (*p* < 0.001) [59]. Sarkar et al. compared multiple ML models for predicting the HCC risk in a cohort of 2247 patients with MASLD. Their study demonstrated that tree-based algorithms, such as gradient boosting, decision tree, and RF, achieved the highest accuracy for predicting HCC, outperforming a probabilistic neural network [60]. Similarly, Audureau et al. applied various ML models to determine the HCC risk in a cohort of 836 patients with chronic HCV based on their sustained virological response (SVR) status. The RF model performed the best, underscoring the potential of ML to enhance HCC risk assessment and facilitate more cost-effective, personalized surveillance programs [61].

Phan et al. evaluated different ML models in a large cohort of patients with viral hepatitis. They found that the CNN model outperformed other models in predicting HCC among patients with chronic hepatitis, achieving an impressive accuracy of 0.980 (AUC = 0.886) [62]. Nam et al. also developed a CNN model that surpassed six older-generation models in predicting the HCC risk in a sample of 424 patients with cirrhosis due to HBV [63].

These studies collectively highlight the evolving landscape of ML models in the prediction and management of HCC. Tree-based algorithms and deep learning models have shown significant promise across various patient cohorts. Their ability to outperform traditional methods such as logistic regression underscores the growing importance of incorporating advanced ML techniques into clinical practice for HCC risk stratification. Moving forward, integrating these models into routine clinical workflows could enable more personalized and proactive management of HCC, potentially improving patient outcomes through timely and targeted interventions.

### 3.2. HCC Diagnosis

CNNs have demonstrated significant utility in analyzing radiology and pathology images for HCC diagnosis by creating feature and pooling layers that emphasize predictive characteristics. Several studies highlight their application and potential.

Chaiteeraakij et al. applied a CNN to ultrasound data from 5444 patients, achieving an HCC detection rate of 82.3% (95% CI: 77.1–87.5) [64]. Mokrane et al. used 1160 imaging features from multiphase CT scans of 178 cirrhotic patients to classify indeterminate liver nodules, identifying a radiological signature with a moderate predictive performance (AUC: 0.66–0.70, sensitivity: 70%, specificity: 54%) [65]. In a larger study, Wang et al. trained two CNN models on CT data from 7512 patients, achieving an AUROC of 0.883 (95% CI: 0.855–0.911) on validation [66]. Zhang et al. used a 3D CNN on MRI data from 267 patients to predict microvascular invasion in HCC, with a combined model yielding an AUC of 0.81, sensitivity of 69%, and specificity of 79% [67]. CNNs have also been applied to monitor tumor progression on CT following locoregional therapy [68]. Kim et al. demonstrated a high sensitivity (87%) and specificity (93%) using CNNs on contrast-enhanced MRI from 549 patients for HCC detection [69]. A meta-analysis by Salehi et al. (44 studies) reported a pooled sensitivity and specificity of 85% (95% CI: 78–89) and 84% (95% CI: 72–91), respectively, across AI algorithms applied to various imaging modalities [70].

Pathology-focused studies also illustrate CNNs’ potential. Chen et al. used CNNs on pathology slides from 377 patients in the Genomics Data Commons Database, training an Inception V3 model to classify whole-slide images. The model achieved an AUC of 0.961 for tumor identification and performed well in tumor grading, with near-perfect agreement with an expert pathologist [71]. Wang et al. trained a CNN on 304 tumors to perform single-cell segmentation, integrating 246 image features to identify histological subtypes and predict survival outcomes [66]. This study underscored CNNs’ role in characterizing tumor microenvironments for personalized treatment.

Lastly, Jang et al. evaluated three CNNs on 20× magnified pathology images, achieving an AUC of 0.995 in differentiating HCC from cholangiocarcinoma and metastatic colon cancer [72].

While these studies underscore CNNs’ efficacy, limitations such as small sample sizes, single-center data, and a lack of external validation limit their generalizability. Variability in imaging protocols and preprocessing techniques also affects reproducibility. Future efforts should focus on multicenter datasets, standardized imaging protocols, and the integration of CNN models into clinical workflows.

### 3.3. Predicting HCC Treatment Response

Numerous ML models have been developed to predict patient survival, tumor recurrence, microvascular invasion, and treatment response in HCC. Radiomics, which can analyze imaging data retrospectively from HCC cohorts, is a prominent technique that has shown a superior performance compared to traditional prognostic tools [73,74]. For example, Sheen et al. created a radiomics nomogram integrating CT-derived Rad scores, tumor staging, alpha-fetoprotein (AFP) levels, and bi-lobar tumor distribution to identify tumors’ refractory to trans-arterial chemoembolization (TACE). This model demonstrated a high predictive accuracy, with AUC values ranging from 0.91 to 0.95 [75]. Similarly, Ji et al. combined contrast-enhanced CT imaging with variables like ALBI grade, AFP levels, cirrhosis status, and tumor margins in ML models to predict HCC recurrence. These models achieved concordance indices (C-index) from 0.633 to 0.699, outperforming traditional staging systems [76]. Qiao et al. developed an ANN model for predicting early HCC outcomes post-hepatectomy in 543 patients, achieving an AUC of 0.855 and surpassing established systems like TNM, BCLC, and IHPBA [77].

DL models can also identify conventional and novel histopathological patterns associated with HCC recurrence after surgical resection or transplantation. By integrating these patterns, DL models generate reliable predictions that improve prognostic accuracy and aid clinical decision making [78,79,80,81]. Saillard et al. used two DL algorithms, SCHMOWDER and CHOWDER, based on whole-slide images (WSI). SCHMOWDER, which focused on pathologist-annotated tumor areas, achieved a C-index of 0.78, while CHOWDER, operating independently, achieved a C-index of 0.75, both outperforming traditional methods. Validation in an independent dataset from The Cancer Genome Atlas (TCGA) confirmed their robust discriminatory power [82]. Chaudhary et al. explored DL-based multi-omics approaches in 360 patients, incorporating RNA sequencing, microRNA sequencing, and methylation data. This model achieved a C-index of 0.68 and identified patient subgroups with significantly different survival outcomes (*p* = 7.13 × 10^−6^). Validation across five external datasets further supported its effectiveness, with C-indices ranging from 0.67 to 0.82 [21].

AI also shows promise in tailoring treatment plans to individual characteristics [83]. ML models can predict liver transplant waitlist dropout rates [84], post-transplant tumor recurrence [85], and graft survival [86]. AI-driven analyses of genetic and molecular data can guide targeted therapies by identifying mutations linked to HCC [87]. Large Language Models (LLMs), like GPT-4, can analyze extensive research and clinical data for evidence-based recommendations. In a recent study, Ge et al. employed Versa GPT-4 to extract data from 1101 imaging reports of 753 HCC patients, achieving an overall accuracy of 0.934. However, challenges remained in extracting more complex data elements [88].

While these studies demonstrate AI’s potential to enhance HCC management, limitations include variability in model performance, a lack of external validation, and dependence on high-quality datasets. Some models, such as those relying on multi-omics data or advanced imaging, may not be feasible in resource-limited settings. Additionally, while LLMs like GPT-4 show promise, their reliability in clinical decision making needs further validation. Future work should focus on integrating AI into clinical workflows, ensuring model interpretability, and validating across diverse populations to maximize their clinical utility.

## 4. Applications of AI in Autoimmune Liver Disease

AI has also demonstrated promise in diagnosing and managing chronic liver disorders such as autoimmune hepatitis (AIH), primary biliary cholangitis (PBC), and primary sclerosing cholangitis (PSC).

Ercan et al. developed a deep learning tool, AI(H), using the Aiforia platform to analyze liver biopsies and accurately identify histological features of AIH [89]. Guadalupi et al. applied a gel-based proteomic approach with ML to analyze salivary samples from patients with AIH, PBC, and healthy controls. Mass spectrometry revealed distinct protein variations, with RF and multidimensional scaling techniques identifying a protein set specific to AIH and PBC. Many of these proteins were linked to immune function and liver fibrosis, offering a non-invasive approach to understanding the pathophysiology of these conditions [90].

In PSC, Singh et al. used MRI data from 169 patients with large-duct PSC to train a decision tree model that identified patients at a high risk for decompensation within a year [91]. Cristoferi et al. and Vuppalanchi et al. employed the MRCP+ software to create 3D models of the biliary tree, generating metrics predictive of PSC disease progression [92,93]. Other studies focused on biomarker identification using ML techniques. Snir et al. analyzed blood samples from 45 PSC patients and 30 controls, identifying 2870 proteins associated with PSC severity and progression to cirrhosis. Elastic net regression highlighted 16 proteins strongly correlated with the enhanced liver fibrosis (ELF) score, mapping to pathways involving CCL24, a protein linked to PSC pathogenesis and cirrhosis risk [94]. Mousa et al. profiled the plasma bile acids in 508 PSC patients and 302 controls using liquid chromatography–tandem mass spectrometry. A gradient boosting model derived the PSC-BAP score based on six bile acid variables, which effectively predicted hepatic decompensation risk and could potentially evaluate targeted therapies [95].

These studies highlight the potential of AI in non-invasive diagnostics, risk stratification, and disease progression monitoring for various liver disorders. However, most of these findings require external validation and replication in larger, diverse cohorts to establish generalizability. While promising, further research is needed to transition these applications from experimental models to widely adopted clinical tools.

## 5. Discussion

The adoption of AI in hepatology can potentially revolutionize the management of complex liver diseases by offering innovative tools for diagnosis, treatment, and personalized care. With the increasing global burden of liver diseases—particularly MASLD and HCC, which are linked to a high morbidity and mortality—AI has emerged as a critical resource for improving early detection and individualizing management strategies. A review of the current literature highlights the significant advantages of various AI techniques in hepatology. AI has demonstrated accuracy in identifying patients with advanced fibrosis and cirrhosis and in predicting clinically significant portal hypertension (CSPH) in chronic liver disease (CLD), often surpassing traditional invasive and non-invasive diagnostic modalities. These capabilities enable early interventions that can improve patient outcomes, such as initiating HCC surveillance or prescribing non-selective beta-blockers to mitigate complications. Additionally, AI supports risk stratification for patients with cirrhosis, helping to identify those with severe CSPH-related complications who may benefit from timely liver transplant referrals. AI also shows promise in tailoring treatment approaches for patients with HCC by accurately predicting treatment responses. This capability enables clinicians to optimize therapeutic strategies and improve patient care. By harnessing its ability to process and analyze complex datasets, AI is becoming an indispensable tool for enhancing clinical decision making and improving outcomes in hepatology.

However, despite significant advancements, integrating AI into the routine clinical management of CLD remains a complex endeavor, requiring strategic planning and addressing several critical challenges. Among these, data limitations and the risk of overfitting stand out. Overfitting, a common issue in developing AI models for hepatology, occurs when models focus on specific patterns in the training data rather than capturing broader, generalizable features. This can lead to a strong performance in development, but a poor validity in diverse clinical settings. Deep learning models trained on small datasets are particularly prone to capturing “noise” or spurious correlations, diminishing their predictive reliability. Strategies such as cross-validation, rigorous external validation with independent datasets, and employing interpretable models when data are limited are essential for mitigating this risk [96,97,98,99,100]. Biases and generalizability issues further complicate AI adoption in hepatology. Many AI models are developed using imbalanced or non-representative datasets, often derived from specific regions or demographic groups. This limits their applicability across global populations with diverse disease etiologies, such as MASLD or alcohol-associated liver disease (ALD) in Western countries versus viral hepatitis in Southeast Asia. Incorporating diverse patient demographics and leveraging bias-aware ML techniques can help to address these disparities and enhance the generalizability of AI models [97,98,101,102,103]. Deep learning algorithms are also frequently regarded as “black boxes” due to their lack of transparency, making it difficult for clinicians to trust their outputs and incorporate them into routine clinical practice. Explainable AI (XAI) approaches, such as Shapley Additive Explanations (SHAPs) and Local Interpretable Model-Agnostic Explanations (LIMEs), offer potential solutions by clarifying decision-making processes. Future efforts must focus on balancing model accuracy with interpretability to align with clinical reasoning and facilitate broader acceptance [104,105,106].

The evolving regulatory landscape of healthcare also poses challenges for the approval and deployment of AI-based tools in hepatology. While regulatory agencies such as the FDA and EMA have issued guidelines for AI-driven medical devices, the lack of standardized protocols for evaluating model safety, efficacy, and equity remains a significant hurdle. Ethical concerns, including data privacy, patient consent, and equitable access, must also be addressed. Transparent and comprehensive policy frameworks, coupled with standardized evaluation metrics and robust validation processes, are necessary to ensure that AI applications meet clinical and ethical standards before their widespread adoption. Engaging patients and the public in the development and deployment of AI tools can enhance transparency and trust in these technologies. Education initiatives that inform patients about AI’s capabilities and limitations, along with feedback mechanisms for data usage, can foster more ethical and patient-centered applications [107,108,109,110,111].

Data integration and interoperability issues further challenge AI implementation. Analyzing diverse data types, such as imaging, genomics, and clinical records, is essential for developing robust AI models. However, inconsistencies in electronic health record (EHR) systems hinder the creation of comprehensive datasets. Advances in multimodal learning frameworks show promise for addressing these challenges, but require refinement to improve integration and performance [112].

Most AI models in hepatology are developed and validated using retrospective datasets, which limits their applicability to real-world clinical settings. Prospective, multicenter studies are crucial to evaluate model performance across diverse patient populations and healthcare environments, enhancing their generalizability and reliability [113,114]. To maintain clinical relevance, AI models must adapt to new data and evolving clinical practices. Continuous learning frameworks allow models to update as new data become available, thereby mitigating the risk of performance degradation over time [115]. However, implementing such systems requires robust monitoring mechanisms to detect shifts in data distribution and ensure a sustained accuracy and reliability in predictions [116].

Finally, the development, implementation, and maintenance of AI systems in healthcare entail substantial costs. Beyond initial investments in technology and infrastructure, ongoing expenses related to data storage, model updates, clinician training, and system integration must be considered. Conducting cost-effectiveness analyses will be vital to determine whether AI tools deliver sufficient clinical benefits to justify their financial impact, particularly in resource-limited settings [117,118].

## 6. Conclusions

While AI holds significant promise for improving the care of patients with chronic liver disease, its widespread implementation continues to face numerous challenges. Overcoming these obstacles will necessitate a multidisciplinary approach, fostering collaboration among clinicians, AI researchers, regulatory agencies, and patient advocacy organizations. By prioritizing transparency, inclusivity, and evidence-based practices, the field of AI in hepatology can progress toward developing clinical applications that are effective, equitable, and trustworthy.

## Figures and Tables

**Table 1:** Summary of the AI techniques referenced in the article.

Name	Brief Description	Advantages	Disadvantages	Examples of Application
Logistic Regression	A statistical model used for binary classification problems. It predicts the probability of a categorical dependent variable.	Simple to understand and implement; computationally efficient.	Limited performance for non-linear problems; assumes linear relationship between dependent and independent variables; sensitive to outliers.	Predicting and diagnosing histological fibrosis stages.
K-Nearest Neighbors (KNNs)	A simple, instance-based learning algorithm that classifies based on most of the nearest neighbors.	Easy to understand and implement, non-parametric, adaptable for small datasets.	High computational cost; inefficient for large datasets; performance degrades with irrelevant features.	Predicting ascites grades based on clinical data.
Support Vector Machine (SVM)	A supervised learning model that classifies data by finding the hyperplane that best separates different classes in the feature space.	Effective in high-dimensional spaces; robust to overfitting; flexible with kernel functions.	Computational complex; sensitive to choice of kernel; not suitable for large datasets; difficult with noisy data.	Predicting fibrosis progression on ultrasound. Screening for cirrhosis and HCC using urine fluorescence spectroscopy.
Decision Trees	Simple models that split data into branches based on decision rules.	Easy to understand and interpret; non-parametric nature; handles feature interactions without requiring explicit specification.	Prone to overfitting; unstable with small data changes; bias towards dominant classes.	Identifying high risk for decompensation in patients with PSC using MRI data.
Ensemble Learning	Combines multiple learning algorithms to obtain better predictive performance than the sum of its parts.	Reduces overfitting risk; improves accuracy.	Can be complex to implement, depends on base model performance.	Predicting variceal bleeding risk using clinical and laboratory data.
Random Forest	An ensemble learning method that builds multiple decision trees and merges their results for more accurate and robust predictions.	High accuracy and robust to noise; reduces overfitting; works with both categorical and numerical data.	Computationally intensive; less interpretable than simpler models; risk of overfitting.	Ruling out cirrhosis using laboratory parameters (FIB-6 index). Developing salivary test to classify autoimmune liver disease.
AdaBoost	Combines multiple weak learners (often decision stumps) into a single strong learner by focusing on misclassified instances.	Works well with weak learners; feature importance; performs well for both classification and regression tasks.	Sensitive to noisy data and outliers; can overfit on small or imbalanced datasets; training is sequential.	Predicting chronic liver disease with feature selection.
Gradient Boosting	Builds models sequentially by optimizing a loss function using gradient descent, where each new model corrects the errors of the previous ones.	High predictive accuracy; effective for complex datasets.	Computationally heavy; can be prone to overfitting if not tuned properly.	Using in studies with large cohorts (e.g., predicting HCC in HBV/HCV patients).
XGBoost	An optimized and scalable implementation of gradient boosting with regularization to prevent overfitting.	Handles missing data (sparsity awareness); reduces overfitting with regularization; efficient on large datasets.	Can be complex to tune for optimal performance; memory-intensive; sensitive to data preprocessing.	Identifying microvascular invasion in HCC using radiomic features.
Light Gradient Boosting Machine (LightGBM)	A gradient boosting framework designed for efficiency and speed with leaf-wise tree growth and histogram-based binning.	Efficient in processing large datasets, fast training speed, high accuracy.	Sensitive to hyperparameters requiring fine-tuning; risk of overfitting with small or imbalanced datasets; may underperform on older hardware systems.	Predicting cirrhosis using laboratory data.
Neural Networks	Algorithms modeled after the human brain, capable of learning from data.	Effective for complex pattern recognition.	High computational cost; can be opaque in decision making.	Differentiating HCC from other liver cancers by using digital whole-slide images.
Artificial Neural Networks (ANNs)	Models mimicking the human brain, using layers of nodes (neurons) for pattern recognition and predictions.	Suitable for complex and non-linear relationships between inputs and outputs; can be applied to various types of data.	Requires large annotated datasets for training; prone to overfitting; black-box nature.	Predicting histological fibrosis stages. Predicting early mortality in HE patients. Predicting early HCC outcomes after partial hepatectomy.
Deep Learning (DL)	Advanced ML models capable of learning high-level abstractions in data and hierarchical representations.	Capable of handling unstructured data and complex patterns; automatic feature extraction; thrive on big data.	Requires substantial computational resources and large datasets for effective training; black-box nature.	Distinguishing ALD cirrhosis using MRI. Predicting CSPH from liver biopsy samples. Identifying AIH features from liver biopsies using Aiforia platform.
Convolutional Neural Networks (CNNs) (e.g., ResNet50, DenseNet121)	Specialized neural networks that use convolutional filters (kernels) to extract spatial features from structured data (e.g., images).	Highly effective for image analysis; automatic feature extraction; translation invariance.	Requires large annotated datasets for training; computationally intensive; limited interpretability.	Detecting cirrhosis-related signals on ECG. Screening and identifying hepatobiliary diseases using ocular images. Predicting CSPH from liver biopsy samples. Diagnosing gastroesophageal varices and predicting rupture risk using endoscopic images (ENDOANGEL).
Large Language Models (LLMs) (e.g., GPT-4)	AI models trained on vast text data for natural language understanding and generation.	Can process extensive literature; assist in data extraction from unstructured sources.	Requires high computational power; may not handle complex tasks as well as specialized models.	Extracting data from unstructured clinical records.
Transfer Learning (e.g., Google’s DeepLabv3+)	Leveraging pre-trained models on new tasks, allowing for faster training and improved performance with less data.	Reduces training time, leverages existing knowledge.	Requires adaptation to specific datasets; potential overfitting if data are not sufficiently similar.	Predicting cirrhosis from CT using automated liver segmentation and image analysis.
Radiomics	Extracts quantitative features from medical images using data characterization algorithms.	Non-invasive; enhanced prognostic and predictive models; quantifies subtle differences; broadly applicable to multiple imaging modalities.	Relies on imaging quality; prone to overfitting without robust feature selection.	Predicting HCC recurrence using CT-derived Rad score. Evaluating tumor characteristics for treatment response and survival prediction.
Pyradiomics	Open-source Python package for extracting radiomic features from 2D and 3D medical imaging.	Standardized feature extraction methods; customizable; comprehensive feature extraction.	Requires accurate segmentation; computationally intensive; steep learning curve.	Extracting radiomics features from US/CT/MRI to predict fibrosis stage and inflammation grading, to assess portal hypertension.

**Table 2:** AI in the management of cirrhosis and its complications.

Study Author(s)	Study Focus	ML Algorithm(s) Used	Study Population	Key Findings
Detection of Advanced Fibrosis and Cirrhosis				
Chang et al. [22]	Predicting fibrosis stages in MASLD patients	LR, RF, Neural Networks	1370 MASLD patients	RF outperformed traditional scoring systems including NFS and FIB-4
Fan et al. [23]	Fibrosis and cirrhosis diagnosis using liver stiffness and aMAP score	ML algorithms	946 patients, biopsy-confirmed MASLD	The ML model had a diagnostic accuracy of 96.8% for advanced fibrosis and 91.2% for cirrhosis
Anushiravani et al. [24]	FIB-6 index for ruling out cirrhosis in hepatitis C	RF	7238 patients with HCV	FIB-6 superior to other indices
Wang et al. [25]	Predicting cirrhosis in hepatitis B using LightGBM and bile acids	LightGBM	506 subjects including normal controls and patients with chronic HBV and cirrhosis of various causes	LightGBM demonstrated superior accuracy compared to non-invasive measures
Orhanbulucu et al. [26]	Predicting CLD	RF, AdaBoost, LightGBM	615 subjects including 540 normal controls and 75 patients with CLD	LightGBM was superior to other models in identifying patients with chronic liver disease
Kuzhippallil et al. [27]	Predicting liver disease using ML models	LightGBM, XGBoost, stacking	583 patients with chronic liver disease (CLD)	LightGBM showed competitive accuracy after feature selection and outlier elimination
Mazumder et al. [28]	Predicting cirrhosis using CT scans	3D-U-Net, DeepLabv3+	351 patients including 96 with cirrhosis and 72 liver transplant recipients	The ML model accurately predicted cirrhosis with lab and demographic data integration
Nowak et al. [29]	Detection of cirrhosis using MRI	Deep transfer learning, ResNet50	713 patients with standard T2-weighted MRI	The ML model outperformed board-certified radiologists in cirrhosis detection
Luetkens et al. [30]	Differentiating alcoholic from non-alcoholic cirrhosis using MRI	ResNet50, DenseNet121	465 patients, MRI images	The ML model accurately differentiated alcohol-related from non-alcohol-related cirrhosis
Wei et al. [31]	Predicting fibrosis staging using MRI radiomics	Pyradiomics software (ITK-SNAP)	332 CLD patients	The radiomics model accurately predicted fibrosis stage and inflammation grade
Destrempes et al. [32]	Assessing steatosis, inflammation, and fibrosis using ultrasound and elastography	RF, bootstrapping	82 patients with CLD	The ML model was more accurate than elastography alone for assessing fibrosis, steatosis and inflammation
Meng et al. [33]	Predicting fibrosis progression in diabetic patients using ultrasound	SVM and ML models	618 patients with diabetes and MASLD	Identified 25 fibrosis-related radiomics features, highlighting five key predictive features
Detection of Clinically Significant Portal Hypertension and High-risk Esophageal Varices				
Bosch et al. [34]	Identifying clinically significant portal hypertension	CNN	221 patients with cirrhosis due to MASLD	The CNN model accurately predicted CSPH using liver biopsy samples
Yu et al. [35]	Grading hepatic venous pressure gradient (HVPG) in cirrhosis	Auto-machine-learning CT radiomics model	429 patients undergoing HVPG measurement and CT scan between 2016 and 2018	The ML model outperformed other non-invasive portal hypertension measures
Noureddin et al. [36]	Predicting CSPH using liver histology scores	Unspecified ML model	143 patients with cirrhosis due to MASLD	The ML model accurately predicted CSPH using liver histology scores
Reinis et al. [37]	Assessing portal hypertension severity in compensated cirrhosis	ML models	1232 patients with compensated advanced chronic liver disease (cACLD)	AUC = 0.589–0.887 for CSPH prediction using lab parameters
Liu et al. [38]	Predicting variceal rebleeding in cirrhosis patients	XGBoost	284 patients with HBV-related cirrhosis	ML model accurately predicted re-bleeding in patients with HBV cirrhosis
Agarwal et al. [39]	Predicting variceal bleeding risk in compensated cirrhosis	XGBoost	828 patients with CLD	ML model accurately predicted variceal bleeding risk in patients with cirrhosis
Bayani et al. [40]	Grading esophageal varices in cirrhosis patients	CatBoost, XGBoost	490 patients with cirrhosis	The ML models outperformed platelet-to-spleen ratio for variceal bleeding risk prediction
Gao et al. [41]	Predicting variceal bleeding using imaging	Various ML models	330 patients with cirrhosis and acute variceal bleeding	Imaging-based ML model outperformed clinical risk scores
Jin et al. [42]	Measuring esophageal varix diameter non-invasively	AI-driven endoscopy model	7 patients with esophageal varices	Precise assessment of variceal size and bleeding risk without direct visualization
Yan et al. [43]	Diagnosing high-risk varices using CT images	Radiomics model	796 patients with cirrhosis and esophageal varices	Radiomics model accurately predicted the presence of high-risk varices, outperforming Baveno VI criteria
Hou et al. [44]	Predicting 1-year variceal bleeding risk using an ANN	ANN	1100 patients with cirrhosis	The ANN model accurately predicted the 1-year risk for variceal bleeding in cirrhotic patients
Chen et al. [45]	Detecting gastroesophageal varices and predicting bleeding risk using a CNN	CNN	3021 patients with cirrhosis and varices on endoscopy	The CNN model outperformed endoscopists in diagnosing varices and identifying risk factors for bleeding
Management of Decompensated Liver Disease				
Wurstle et al. [46]	Identifying infected ascites using clinical data	ML models	700 episodes of patients with decompensated liver cirrhosis undergoing abdominocentesis	The ML model had a high negative predictive value for infected ascites, reducing reliance on paracentesis
Hatami et al. [47]	Predicting ascites grades in cirrhosis patients	KNN, SVM, neural networks classification system	492 patients with cirrhosis	KNN model achieved the highest accuracy for predicting ascites severity
Yang et al. [48]	Predicting early mortality in hepatic encephalopathy (HE) patients	Weighted random forest (WRF), SVM, and weighted SVM (WSVM)	1256 patients with cirrhosis	WRF model was most accurate at predicting the incidence of HE in patients
Zhang et al. [49]	Predicting mortality in HE	Artificial neural network (NNET) model	601 patients with HE from Medical Information Mart for Intensive Care (MIMI)-IV database	ANN model had the highest accuracy for predicting mortality in patients with HE

ML (Machine Learning), DL (Deep Learning), RF (Random Forest), CNN (Convoluted Neural Network), ANN (Artificial Neural Network), KNN (K-Nearest Neighbor), LLM (Large Language Model), SVM (Support Vector Machine), DT (Decision Tree), LR (Logistic Regression), and CLD (chronic liver disease).

**Table 3:** AI in the management of hepatocellular carcinoma.

Author(s)	Study Focus	ML Algorithm(s) Used	Study Population	Key Findings
HCC Risk Stratification				
Singal et al. [55]	Predict HCC risk in patients with cirrhosis using an ML model	RF	442 patients with cirrhosis	The ML model was more accurate in predicting HCC in compared to other predictive models
Kim et al. [56]	Predict HCC risk in patients with HBV using an ML model	GBM	6051 Korean and Caucasian patients with chronic HBV	The GBM model was more accurate in predicting HCC risk compared to other predictive models
Sato et al. [57]	Compare the accuracy of various ML models to predict HCC risk in patients with CLD	SVM, RF, neural networks, and GBM	4242 patients with CLD of various etiologies	Gradient boosting outperformed other ML algorithms
Wong et al. [58]	Compare the accuracy of various ML techniques to predict HCC risk in patients with chronic hep B and hep C	AdaBoost, RF, RR, LR, and DT	124,006 patients from Hong Kong with chronic HBV or HCV	Ridge regression and random forest outperformed other models in the validation cohort
Lin et al. [59]	Use an elastography-based ML model to predict HCC in patients with CLD	Unspecified ML algorithm	5155 Korean patients with CLD	The ML algorithm outperformed other HCC predictive models
Sarkar et al. [60]	Compare the performance of various ML algorithms to predict HCC risk in patients with MASLD	GBM, neural networks, RF, DT	2247 patients with MASLD	GBM model was the most accurate at predicting HCC, followed by the RF model
Audureau et al. [61]	Compare ML models for predicting HCC patients with HCV	RF, DT	836 patients with HCV including 160 patients with biopsy proven HCC	ML algorithms enhanced HCC risk prediction enabling more precise and individualized assessments
Phan et al. [62]	Compare deep learning models for detecting HCC in patients with viral hepatitis	Various DL models	1 million patients with viral hepatitis from the NHIRD database in Taiwan	Among the various DL models, convolutional neural network (CNN) had the highest accuracy
Nam et al. [63]	Compare a DL model using CNN to traditional HCC prediction models in patients with HBV cirrhosis	CNN	424 Korean patients with cirrhosis due to chronic HBV	The DL model outperformed traditional HCC prediction models and successfully classified patients into low and high risk for HCC
HCC Diagnosis				
Chaiteeraakij et al. [64]	Develop an AI assisted system that can successfully detect and classify focal liver lesions	CNN (YOLOv5)	5444 patients from Thailand with 26,288 US images	The AI system was able to distinguish benign from malignant lesions with high sensitivity and specificity
Mokrane et al. [65]	Identify with indeterminate liver lesions at high risk for HCC using ML	Radiomics, RF, SVM, KNN	178 patients with cirrhosis and indeterminate liver lesions (138 patients had biopsy prove HCC)	The ML model was able to identify a subset of patients who were at high risk for HCC
Wang et al. [66]	Use a DL model to identify distinct tumor subtypes from WSI and assess their association with prognosis	CNN	304 patients from the Cancer Genome Atlas HCC cohort	The DL model identified 3 distinct histological subtypes with variable prognoses based on several features
Zhang et al. [67]	Develop a radiomics model to identify HCC patients with microvascular invasion	Radiomics	267 patients with HCC	The radiomics model effectively distinguished between MVI and non-MVI HCC
Lim et al. [68]	Detect tumor progression after ablation for hepatocellular carcinoma (HCC) using a CNN model	CNN	74 patients with HCC who underwent ablation and surveillance CT scans	The CNN model accurately identified local tumor progression on surveillance CT scans following tumor ablation
Kim et al. [69]	Identify HCC using a CNN model and compare its performance to that of radiologists	CNN	549 patients with HCC who underwent resection	The CNN model was faster and more accurate at detecting smaller lesions compared to radiologists with lesser experience
Salehi et al. [70]	Evaluate various AI models for the detection of HCC	Various ML techniques	NR	AI can serve as a diagnostic aid for detecting HCC
Chen et al. [71]	Use a CNN model to identify benign and malignant lesions from WSI	CNN	377 patients	The CNN model was very accurate compared to an experienced pathologist
Jang et al. [72]	Differentiate HCC from other types of liver lesions using an ML model	CNN	NR	The CNN model accurately distinguished between HCC and other tumor types
HCC Treatment Response				
Levi-Strauss et al. [73]	Review article on role of Radiomics in the management of HCC	Radiomics	Not Applicable	Radiomics can help diagnose HCC and predict treatment response
Lai et al. [74]	Systematic review on the prognostic role of AI in HCC	Radiomics, ANN, SVM	25,073 patients from 9 studies	All AI models outperformed more traditional predictors of survival in HCC patients
Sheen et al. [75]	Predict tumors refractory to TACE using an ML model	Radiomics	80 patients with HCC treated with TACE	The radiomics model accurately identified tumors that were refractory to TACE
Ji et al. [76]	Predict HCC recurrence after resection using an ML model	Radiomics	470 patients who underwent CT scans and curative resection for solitary HCC	The radiomics model identified a three-feature signature that demonstrated favorable prediction of HCC recurrence
Qiao et al. [77]	Predict post resection survival using an ML model compared to traditional staging systems	ANN	829 patients who underwent resection for HCC	The ANN model outperformed traditional staging systems in predicting post resection survival
Song et al. [78]	Identify patients with MVI using a CNN model compared to a radiomics model	CNN, Radiomics	601 patients with HCC including 225 patients with MVI on pathology specimens	The DL model in combination with clinical parameters was most effective at predicting MVI
Zhang et al. [79]	Predict MVI in HCC patients using a CNN model	DL, CNN	270 patients with confirmed HCC	CNN model demonstrated low to moderate accuracy in detecting MVI, possibly due to small sample size
Jiang et al. [80]	Identify MVI in HCC patients using two AI models	Radiomics, CNN, XGBoost	405 patients with HCC (220 with confirmed MVI)	Both models accurately identified patients with MVI and predicted tumor margin status
Calderaro et al. [81]	Review article on the role of AI in diagnosis and management of gastrointestinal (GI) and hepatobiliary cancer	Not specified	Not applicable	AI can significantly impact the management of GI and hepatobiliary cancer through analysis on histopathology images
Saillard et al. [82]	Predict survival in HCC patients who underwent resection using 2 DL models	DL	194 patients with HCC who underwent resection	Both DL models were able to accurately predict survival
Chaudhary et al. [21]	Predict survival outcomes in patients with HCC using a DL model	DL, SVM	360 patients with HCC from the Cancer Genome Atlas	The model identified two patient subgroups with significantly different survival outcomes
Choi et al. [83]	Use ML to guide treatment selection and predict survival in HCC patients	RF	1021 patients with HCC	The model performed well in recommending treatments and predicting overall survival
Kwong et al. [84]	Predict waitlist drop off among listed HCC patients using an ML model	RF	18,920 US patients with HCC and listed for a transplant	The ML model accurately predicted dropout risk at 3, 6, and 12 months
Nam et al. [85]	Predict HCC recurrence after liver transplantation (LT) using an ML model	Neural networks	563 patients who underwent LT for HCC	The ML model outperformed conventional models for predicting HCC recurrence after LT
Briceño et al. [86]	Enhance donor–recipient matching in LT using an ML model	ANN	1003 LT recipients from Spain	Two ANN models significantly outperformed traditional metrics in predicting survival after LT
Elmas et al. [87]	Analyze proteogenomic data from HCC patients using ML to identify potential therapeutic targets	KNN	260 patients with HBV and HCC	The study identified several potential treatment targets demonstrating the potential of proteomics approaches in advancing precision medicine in HCC
Ge et al. [88]	Extract structured data from 1101 HCC imaging reports using a large language model (LLM)	LLM	753 patients with HCC	The LLM performed exceptionally well on simple tasks like identifying macrovascular invasion but was less effective for more complex tasks

ML (Machine Learning), DL (Deep Learning), RF (Random Forest), CNN (Convoluted Neural Network), ANN (Artificial Neural Network), KNN (K-Nearest Neighbor), LLM (Large Language Model), SVM (Support Vector Machine), DT (Decision Tree), WSI (Whole Slide Images), and GBM (Gradient Boosting Machine).
